# Changes in antimicrobial utilization during the coronavirus disease 2019 (COVID-19) pandemic after implementation of a multispecialty clinical guidance team

**DOI:** 10.1017/ice.2020.1291

**Published:** 2020-10-26

**Authors:** Milner B. Staub, Ronald M. Beaulieu, John Graves, George E. Nelson

**Affiliations:** 1 Vanderbilt University Medical Center, Division of Infectious Diseases, Nashville, Tennessee; 2 Veterans Health Administration, Tennessee Valley Healthcare System, Geriatric Research Education and Clinical Center, Nashville, Tennessee; 3 Vanderbilt University School of Medicine, Department of Medicine, Nashville, Tennessee

## Abstract

**Objective:**

Evaluate changes in antimicrobial use during COVID-19 and after implementation of a multispecialty COVID-19 clinical guidance team compared to pre–COVID-19 antimicrobial use.

**Design:**

Retrospective observational study.

**Setting:**

Tertiary-care academic medical center.

**Participants:**

Internal medicine and medical intensive care unit (MICU) provider teams and hospitalized COVID-19 patients.

**Methods:**

Difference-in-differences analyses of antibiotic days of therapy per 1,000 patient days present (DOT) for internal medicine and MICU teams treating COVID-19 patients versus teams that did not were performed for 3 periods: before COVID-19, initial COVID-19 period, and after implementation of a multispecialty COVID-19 clinical guidance team which included daily, patient-specific antimicrobial stewardship recommendations. Patient characteristics associated with antibiotic DOT were evaluated using multivariable Poisson regression.

**Results:**

In the initial COVID-19 period, compared to the pre–COVID-19 period, internal medicine and MICU teams increased weekly antimicrobial use by 145.3 DOT (95% CI, 35.1–255.5) and 204.0 DOT (95% CI, −16.9 to 424.8), respectively, compared to non–COVID-19 teams. In the intervention period, internal medicine and MICU COVID-19 teams both had significant weekly decreases of 362.3 DOT (95% CI, −443.3 to −281.2) and 226.3 DOT (95% CI, −381.2 to –71.3). Of 131 patients hospitalized with COVID-19, 86 (65.6%) received antibiotics; no specific patient factors were significantly associated with an expected change in antibiotic days.

**Conclusions:**

Antimicrobial use initially increased for COVID-19 patient care teams compared to pre–COVID-19 levels but significantly decreased after implementation of a multispecialty clinical guidance team, which may be an effective strategy to reduce unnecessary antimicrobial use.

Severe acute respiratory syndrome coronavirus 2 (SARS-CoV-2), the pathogen responsible for coronavirus disease 2019 (COVID-19), was identified in December 2019, sparking a global pandemic.^1,2^ This viral pandemic stands to exacerbate an already escalating antimicrobial resistance epidemic by increasing antimicrobial use, the primary driver of antimicrobial resistance.^3^ Descriptive studies have evaluated COVID-19 patient demographics, medical conditions, laboratory parameters, and risk factors for severe disease.^4–13^ Data exploring bacterial coinfection and secondary infection are emerging.^14–16^

The specter of bacterial superinfection looms based on seasonal and pandemic influenza experience.^17,18^ Inconsistent definitions, lack of detailed descriptions, and inclusion of few, often clinically different patients, preclude definitive conclusions to inform appropriate antimicrobial guidance for COVID-19 patients.^14^ Currently, data suggest low COVID-19 bacterial superinfection rates. Meta-analyses of hospitalized COVID-19 patients reported 62 of 806 patients (8%)^19^ and 153 of 2,183 patients (7%)^20^ had confirmed bacterial coinfection. Some studies have demonstrated no confirmed secondary infections.^21,22^ The true prevalence of COVID-19 and bacterial coinfection is unknown, potentiating unnecessary empiric bacterial coverage.^6,23–26^ The benefits of antimicrobial use reduction must be balanced with risk of serious bacterial secondary infections, especially in critically ill patients where mortality rates may approach 50%.^6^

Additional US COVID-19 epidemiologic data are emerging, but antimicrobial use data remain scant globally.^22,27–30^ Estimates range widely, but often >90% of hospitalized COVID-19 patients receive antimicrobials.^14^ Antimicrobial use is typically higher in critically ill COVID-19 patients; it is empiric rather than culture directed; and it consists of broad-spectrum coverage.^19,31,32^ Beyond adversely impacting global antimicrobial resistance, unnecessary antimicrobial use may potentiate antibiotic-resistant infections in COVID-19 patients,^33^ described in limited case reports.^34,35^ Although emerging data can improve understanding of bacterial coinfection and antimicrobial use, there is a need to develop antimicrobial stewardship processes in COVID-19 management.^29,33,36,37^

Using a differences-in-differences (DiD) design in this retrospective study of COVID-19 patients hospitalized from March 1 through May 15, 2020, we aimed to evaluate how antimicrobial use among internal medicine and medical intensive care unit (MICU) provider teams changed before and after the COVID-19 pandemic and whether implementation of a multispecialty COVID-19 clinical guidance team (“COVID-19 huddle”) that included antimicrobial stewardsip recommendations influenced antimicrobial use.

## Methods

This single-center retrospective observational study was conducted at Vanderbilt University Medical Center (VUMC), an academic, tertiary, acute-care hospital in Nashville, Tennessee. The study period was separated into a pre–COVID-19 period (December 1 to February 29) and 2 post–COVID-19 periods (March 1–21 and March 22–May 15). The initial post–COVID-19 period started the week of the first confirmed Tennessee COVID-19 patient (March 4, 2020). The second post–COVID-19 period started with implementation of the COVID-19 huddle (March 24).

Patients eligible for inclusion in secondary outcome analysis were ≥18 years old and had been admitted for >12 hours to internal medicine or MICU teams with a positive SARS-CoV-2 real-time polymerase chain reaction (RT-PCR) assay from nasal, oropharyngeal, or nasopharyngeal swab specimens between March 1, 2020 and May 15, 2020. Only the initial admission after a positive SARS-CoV-2 assay was included. Demographic data, comorbid conditions, microbiology data, and medication data were collected through automated electronic medical record (EMR) chart extraction based on ICD-10 codes billed by primary and consulting teams and from the patient’s EMR pre-existing problem list.

At VUMC, confirmed and suspected COVID-19 patients were cohorted and placed under the care of select COVID-19 teams to optimize efficiency, personal protective equipment resources, and management standardization. Two pre-existing internal medicine hospitalist teams were designated to admit COVID-19 patients (internal medicine COVID team). Up to 5 pre-existing hospitalist internal medicine teams continued to admit non–COVID-19 acute-care patients (internal medicine non–COVID-19 team). One pre-existing, nonteaching MICU team was designated to admit critically ill COVID-19 patients (MICU COVID-19 team). Up to 3 pre-existing MICU teams continued to admit non–COVID-19 critical care patients (MICU non-COVID team). Daily antimicrobial use data for individual teams were aggregated by team type (internal medicine and MICU, COVID-19 and non–COVID-19) and weekly antimicrobial use changes were evaluated over the 3 periods using DiD analysis.

### Care team and patient outcomes

The primary outcome was the difference in change in weekly antimicrobial use for COVID-19 teams and change in weekly antimicrobial use for non–COVID-19 teams, calculated by comparing teams to their own performance, separately for internal medicine and MICU teams, during 3 periods: before COVID-19; during COVID-19 before implementation of a COVID-19 huddle; and during COVID-19 after the implementation of a COVID-19 huddle. The secondary outcome was to evaluate whether certain patient characteristics were associated with increased antimicrobial use duration for COVID-19 patients.

### Antibiotic use

Antimicrobial use was calculated as days of therapy per 1,000 patient days present (DOT per 1,000 days).^38^ A day of therapy was counted as any amount of an antibiotic administered in a calendar day to a patient. Patient days present were the number of patients admitted to a service team anytime throughout a day. Data were obtained from VUMC antimicrobial stewardship program’s validated antibiotic surveillance dashboard. Only antibiotics were included in antimicrobial use calculation. All antifungals, antivirals, and hydroxychloroquine were excluded. Given the high variability of daily antimicrobial use, weekly antimicrobial use was reported; however, daily DOT per 1,000 days for each team were entered into the DiD model to yield the most accurate results when using fixed effects at the week and team-type levels.

### Institutional guidelines and multispecialty clinical guidance team (“COVID-19 huddle”) interventions

During the week of March 8, 2020, the VUMC infectious diseases SARS-CoV-2 pharmacotherapeutics working group created COVID-19 clinical guidance, available to all providers, initially by website but updated and e-mailed weekly starting March 16. On March 22, VUMC implemented a “COVID-19 huddle” composed of pulmonary critical care, infectious diseases, palliative care, nursing and social work leaders who met daily to discuss all admitted COVID-19 patients. Assigned providers for COVID-19–specific teams and investigators representing ongoing COVID-19 clinical trials at VUMC also attended. Each patient was discussed, including current medications, culture and lab results, care plan, trial eligibility, and discharge plans. The primary provider made final clinical care decisions. Meetings lasted 1–1.5 hours but shortened to ~30 minutes as meetings became routine.

Initially, 2 rotating infectious diseases physicians (later expanding to weekly rotation of all infectious diseases faculty) participated, making case-by-case antibiotic recommendations. Using VUMC clinical guidelines and infectious disease expertise, they helped providers feel comfortable de-escalating or stopping antibiotics when test results suggested low risk of bacterial pneumonia. The VUMC guidelines beginning April 13 (3 weeks after huddle implementation) specifically recommended stopping antibiotics in patients with low or relatively low procalcitonin levels (defined as <0.25 µg/L and 0.25–0.5 µg/L, respectively). On-site, rapid procalcitonin testing became available April 15. For acute-ward COVID-19 patients with procalcitonin >0.5 µg/L or high concern for cobacterial pneumonia awaiting further test results, ceftriaxone and azithromycin or levofloxacin were recommended. The guidelines never recommended the use of azithromycin for treatment of COVID-19.

## Statistical analysis

### Difference-in-differences analyses

We conducted a DiD analysis comparing differential changes in weekly antimicrobial use among COVID-19 and non–COVID-19 internal medicine hospitalist and MICU teams before and after the 2 COVID-19 periods defined in the previous section. Owing to approximately normal distribution of the primary outcome, we utilized ordinary least squares regression. All regressions included fixed effects for admitting team to account for time-invariant attributes of teams that could confound outcome comparisons. We also included fixed effects for week of service to capture secular time trends. Results were reported in weekly DOT per 1,000 days with 95% confidence intervals (CIs).

To account for potential confounding in antimicrobial use rate changes for COVID-19 teams from changes in azithromycin use, which may have been higher early on because of reports of possible benefit from azithromycin and hydroxychloroquine for COVID-19 treatment^24^ (although this was never a VUMC recommended combination), a secondary analysis evaluating DiD of azithromycin antimicrobial use among COVID-19 versus non–COVID-19 teams in the pre– and post–COVID-19 periods was performed. Azithromycin antimicrobial use was compared to ceftriaxone antimicrobial use for internal medicine teams and to cefepime antimicrobial use for MICU teams. Ceftriaxone and cefepime were chosen as comparators based on their consistent, frequent use at VUMC for empiric pneumonia coverage along with azithromycin in acute-ward and intensive care patients, respectively. If there was no difference between azithromycin and ceftriaxone or cefepime use, respectively, we deduced that most azithromycin antimicrobial use was for empiric bacterial pneumonia. However, if there was a difference, then we would consider the difference potentially attributed to azithromycin used specifically to treat COVID-19.

**Fig. 1.**
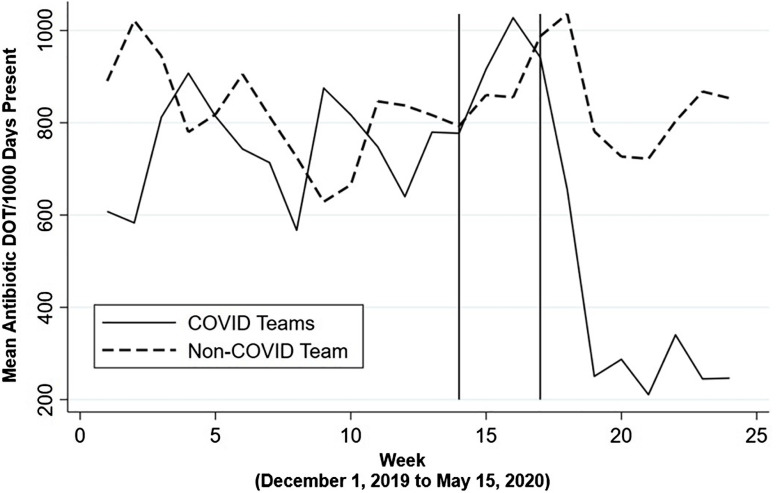
Weekly mean antibiotic use (DOT per 1,000 days present) for non–COVID-19 versus COVID-19 admitting internal medicine teams. This figure shows individual daily team antibiotic use aggregated by team type and averaged weekly. Vertical solid line represents March 1, 2020 (week 14), the first week a COVID-19 positive patient was admitted to Vanderbilt University Medical Center. Second vertical line represents the week of March 24, 2020 (week 17), the first week a multispecialty COVID-19 team convened to consult on admitted COVID-19 patients. Note. DOT, days of therapy.

**Fig. 2.**
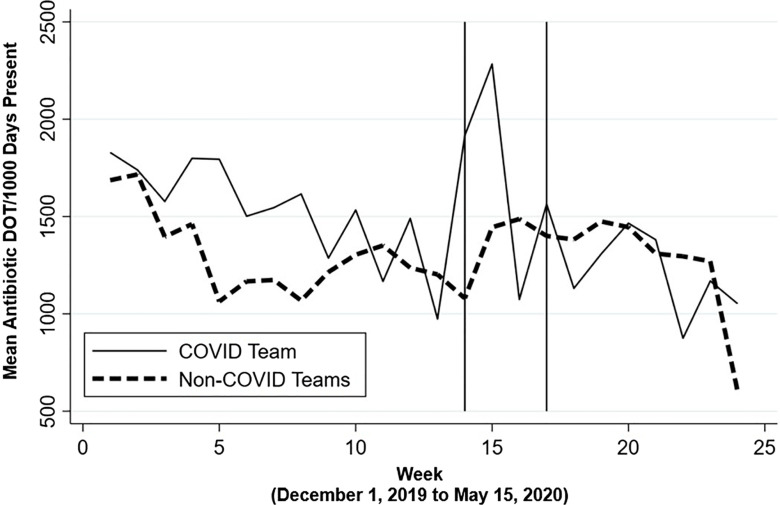
Weekly mean antibiotic use (DOT per 1,000 days present) for non–COVID-19 versus COVID-19 admitting medical intensive care unit teams. This figure shows individual daily team antibiotic use aggregated by team type and averaged weekly. Vertical solid line represents March 1, 2020 (week 14), the first week a COVID-19 positive patient was admitted to Vanderbilt University Medical Center. Second vertical line represents the week of March 24, 2020 (week 17), the first week a formal multispecialty COVID-19 team convened to consult on admitted COVID-19 patients. Note. DOT, days of therapy.

Daily team antimicrobial use was aggregated and averaged weekly by designated team type and graphed in the Figures 1 and 2 and Supplementary Figures 1 and 2 (online) to visualize the measured changes that were analyzed using DiD.

### Confirmed COVID-19 patient antimicrobial use analysis

Descriptive patient summary data were reported as counts and percentages for categorical variables and mean (± standard deviation) for continuous variables.

A multivariable Poisson regression was used to evaluate the association between specific patient characteristics and the expected change in antibiotic DOT per individual patient days present. Antibiotic DOT was defined as days in which a patient received an antibiotic. Days present was defined as number of days the patient was hospitalized up to 14 days after admission or after a COVID-19–positive test result, whichever came later. For continuous antibiotics started within this period and extending beyond 14 days, patient days were extended through final antibiotic day. Results were reported as the expected change in log antibiotic DOT per days present (Poisson coefficient) with corresponding incident rate ratios and 95% confidence intervals. Analyses were performed using STATA/MP version 16.1 software (StataCorp, College Station, TX).

This study was approved by the VUMC Institutional Review Board.

## Results

### Team-based antibiotic use

Compared to the pre–COVID-19 period, internal medicine COVID-19 teams had an initial increase in weekly antimicrobial use of 145.3 DOT per 1,000 days (95% CI, 35.1–255.5) in the first post–COVID-19 period when compared to non–COVID-19 internal medicine teams, adjusted for individual team and week (Fig. 1). After implementation of the COVID-19 huddle, the COVID-19 internal medicine teams showed a significant reduction in weekly antimicrobial use of 362.3 DOT per 1,000 days (95% CI, −443.3 to −281.2) from pre–COVID-19 antimicrobial use, adjusted for individual team and week (Fig. 1).

Similarly, when compared to the pre–COVID-19 period, the COVID-19 MICU team also showed an initial weekly increase in antimicrobial use of 204.0 antibiotic DOT per 1,000 (95% CI, −16.9 to 424.8) days in the first 3 weeks of the post–COVID-19 period compared to non–COVID-19 MICU teams, adjusted for individual team and week, although this increase was not statistically significant (Fig. 2). This increase was also followed by a significant reduction in weekly antimicrobial use of 226.3 antibiotic DOT per 1,000 days (95% CI, −381.2 to −71.3) from pre–COVID-19 antimicrobial use for COVID-19 compared to non–COVID-19 MICU teams after implementation of the COVID-19 huddle (Fig. 2).

DiD evaluation of azithromycin use in the pre–COVID-19 period versus the first 3 weeks of admitting COVID-19 patients showed a significant weekly increase of 48.7 DOT per 1,000 days (95% CI, 21.7–75.7) compared to a significant weekly increase of 47.9 DOT per 1,000 days (95% CI, 12.6–83.2) for ceftriaxone use for internal medicine teams. In the second post–COVID-19 period, there was a significant decrease in azithromycin weekly antimicrobial use of 58.2 DOT per 1,000 days (95% CI, −78.0 to −38.3) and a nonstatistically significant increase in ceftriaxone weekly antimicrobial use of 13.6 DOT per 1,000 days (95% CI, −12.4 to 39.6) present, adjusted for week and team (Supplementary Fig. 1 online). Azithromycin use and cefepime use for MICU teams over the same period showed no statistically significant difference in DOT per 1,000 days for the first 3 weeks or the later 8 weeks of the defined COVID-19 period compared to the pre–COVID-19 period when adjusted for week and team (Supplementary Fig. 2 online).

### Confirmed COVID-19 patient antimicrobial use analysis

Of 238 potential COVID-19 patient hospitalizations, 131 (55.0%) unique patients with confirmed COVID-19 results were admitted to either internal medicine or MICU teams from March 1 to May 15, 2020, and were included for analysis. The average age was 56.0 years (±17.4 SD), and 52 of 131 (39.7%) were female. Also, 54 (41.2%) had acute respiratory failure, hypoxia or dyspnea recorded; 43 (32.8%) patients required ICU care; and 86 (65.6%) received antibiotics. A history of cardiovascular conditions (n = 66, 50.4%) and chronic metabolic conditions (n = 49, 37.4%) were the most common underlying patient conditions (Table 1).

**Table 1.** COVID-19 Patients Admitted to Internal Medicine or Medical Intensive Care Unit Teams from March 1 to May 15, 2020

Characteristic	COVID-19–Positive Patients (N = 131), No. (%)
Sex, female	52 (39.7)
Age, mean y (SD),	56.0 (17.4)
BMI, mean (SD)^a^	29.6 (6.3)
Time to admission from COVID-19 test, mean d (SD)	1.7 (3.0)
Received antibiotics	86 (65.6)
Antibiotic duration post-COVID-19 result, mean d (SD)^b^	4.4 (3.5)
MICU care	43 (32.8)
Other documented infection^c^	15 (12.2)
Dementia	8 (6.1)
Smoking history	4 (3.1)
Cancer	11 (8.4)
Sepsis or shock	12 (9.2)
Stroke history	7 (5.3)
Death	13 (9.9)
Hepatitis, current or history^d^	23 (17.6)
Substance abuse^e^	6 (4.6%)
Cardiovascular problems, chronic^f^	66 (50.4)
Pulmonary problems, chronic^g^	22 (16.8)
Immunocompromised^h^	9 (6.9)
Acute respiratory issues^i^	54 (41.2)
Metabolic problem, chronic^j^	49 (37.4)
Renal problem, acute or chronic^k^	24 (18.3)

Note. SD, standard deviation; BMI, body mass index (calculated as weight in kilograms divided by height in meters squared); MICU, medical intensive care unit.

a In kg/m^2^ (n=24).

b Mean antibiotic duration calculated for those who received any antibiotic (n=86).

c Includes cellulitis, abscess, osteomyelitis, bacteremia, urinary tract infection, endophthalmitis.

d Includes current and history of hepatitis A, B or C, alcoholic and nonalcoholic hepatitis, and elevated transaminases.

e Includes drug and alcohol abuse.

f Includes hypertension, arrythmia other than sinus tachycardia or sinus bradycardia, congestive heart failure, and coronary artery disease.

g Includes asthma, chronic obstructive pulmonary disease, and obstructive sleep apnea.

h Includes human immunodeficiency virus, chronic variable immune deficiency, hypogammaglobulinemia, history of organ transplant and rheumatological diseases including lupus and rheumatoid arthritis for which immunosuppression is used.

i Includes documented hypoxia, dyspnea or tachypnea without respiratory failure and documented respiratory failure.

j Includes diabetes, prediabetes, and hyperlipidemia or hypercholesterolemia.

k Includes acute renal injury, chronic kidney diseases, and end-stage renal disease with or without dialysis.

In multivariable Poisson regression analysis of COVID-19 patients, no specific patient factors were had a statistically significant association with an increase or decrease in expected antibiotic DOT per days present (Table 2).

**Table 2.** Expected Change in Antibiotic Days per Days Present Based on Patient Characteristics

Characteristic	Expected Increase in Log Antibiotic Days per Days Present per Unit Change	Incident Rate Ratio	95% Confidence Intervals
Age	−0.002	1.00	0.98–1.02
Sex, female	0.05	1.05	0.54–2.01
Time to admit after COVID-19 test	−0.01	0.99	0.89–1.09
Death	0.72	2.05	0.60–6.97
MICU care	0.30	1.35	0.64–2.85
Other documented infection^a^	0.81	2.25	0.93–5.47
Dementia	−0.57	0.56	0.12–2.73
Cancer	0.58	1.79	0.73–4.35
Sepsis or shock	−0.03	0.97	0.29–3.25
Stroke history	−0.14	0.87	0.21–3.63
Smoker, past or current	0.73	2.08	0.35–12.48
Hepatitis, current or history^b^	0.31	1.36	0.59–3.18
Substance abuse^c^	−1.03	0.36	0.03–3.75
Cardiovascular problems, chronic^d^	−0.30	0.74	0.35–1.57
Pulmonary problems, chronic^e^	0.05	1.05	0.44–2.54
Immunocompromised^f^	0.10	1.10	0.37–3.31
Acute respiratory problem^g^	−0.15	0.86	0.42–1.75
Metabolic problem, chronic^h^	0.09	1.09	0.52–2.30
Renal problem, acute or chronic^i^	−0.21	0.81	0.33–2.01

Note. MICU, medical intensive care unit.

a Includes cellulitis, abscess, osteomyelitis, bacteremia, urinary tract infection, endophthalmitis.

b Includes current and history of hepatitis A, B or C, alcoholic and nonalcoholic hepatitis, and elevated transaminases.

c Includes drug and alcohol abuse.

d Includes hypertension, arrythmia other than sinus tachycardia or sinus bradycardia, congestive heart failure, and coronary artery disease.

e Includes asthma, chronic obstructive pulmonary disease, and obstructive sleep apnea.

f Includes human immunodeficiency virus, chronic variable immune deficiency, hypogammaglobulinemia, history of organ transplant, and rheumatological diseases including lupus and rheumatoid arthritis for which immunosuppression is used.

g Includes documented hypoxia, dyspnea, or tachypnea without respiratory failure and documented respiratory failure.

h Includes diabetes, pre-diabetes, and hyperlipidemia or hypercholesterolemia.

i Includes acute renal injury, chronic kidney diseases, and end-stage renal disease with or without dialysis.

## Discussion

The COVID-19 pandemic may worsen existing antimicrobial resistance through unnecessary antimicrobial use. Currently, antimicrobial use data during COVID-19 are scarce. The potential collateral damage from unnecessary antimicrobial use and promotion of antimicrobial resistance requires investigation and antimicrobial stewardship intervention development to optimize patient outcomes and mitigate downstream effects.^14,19,33^ Here, we describe a brief, initial increase in antimicrobial use for teams caring for COVID-19 patients compared to those teams’ antimicrobial use prior to COVID-19 followed by significant antimicrobial use reduction over time after implementing facility guidelines reinforced by a multispecialty COVID-19 clinical guidance team that included infectious diseases and antimicrobial stewardship expertise.

The overlap between COVID-19 and bacterial pneumonia clinical symptoms and often limited COVID-19 diagnostics availability created clinical uncertainty, likely contributing to antimicrobial overuse. The paucity of data on secondary bacterial infection risk and reliance on prior influenza pandemic data further complicated antibiotic management.^14,17,18^ The implementation of a scheduled, multispecialty, daily COVID-19 huddle with evidence-based facility guidelines allowed streamlining of resources and knowledge to support frontline physicians and optimize COVID-19 patient clinical care, including safely de-escalating and stopping antibiotics.

Secondary bacterial infection rate data are emerging, but studies on antimicrobial use remain limited.^19,20^ Although nearly two-thirds of COVID-19–confirmed patients (65.6%) received an antibiotic, evaluation of COVID-19 internal medicine and MICU teams demonstrated significant reductions in antimicrobial use compared to time before and early in the pandemic, consistent with appropriate treatment of a viral pathogen. Specific analyses of antibiotics used primarily for patients hospitalized with pneumonia (ie, azithromycin compared to ceftriaxone or cefepime in acute ward and ICU teams, respectively) showed an increase in both azithromycin and ceftriaxone in the initial period, consistent with empiric coverage of bacterial coinfection.

After the implementation of the COVID-19 huddle, which universally discouraged azithromycin use for COVID-19 treatment alone and encouraged antibiotic cessation in patients whose symptoms could be attributed to COVID-19, there was a significant decrease in azithromycin use for COVID-19 teams versus non–COVID-19 teams compared to pre–COVID-19 use. Ceftriaxone use did not significantly change for COVID-19 versus non–COVID-19 teams from pre–COVID-19 antimicrobial use rates. It is possible that targeting cessation of azithromycin use for COVID-19 treatment drove significant antimicrobial use reduction in COVID-19 internal medicine teams, but the magnitude of change in azithromycin is less than the overall antimicrobial use reduction for COVID-19 internal medicine teams (−58.2 vs −362.3 DOT per 1,000 days), suggesting that the reduction in the use of several antibiotics contributed. For MICU teams, there was no significant difference in azithromycin or cefepime use among COVID-19 versus non–COVID-19 teams compared to the pre–COVID-19 era. These findings support the belief that the COVID-19 huddle was able to reduce antimicrobial use in COVID-19 patients under the care of the internal medicine team and prevent increased antimicrobial use in COVID-19 patients under the care of the MICU team, consistent with appropriate stewardship for a virally mediated infection.

The urgent need for antimicrobial stewardship during the pandemic has been recognized, but no antimicrobial stewardship interventions have been described.^19^ The observation of antimicrobial use reductions observed in this study does not invalidate concerns of broad overuse, especially considering the challenges with resourcing and implementing antimicrobial stewardship effectively. Aggressive inclusion of infectious diseases and antimicrobial stewardship embedded within treatment teams may reduce antimicrobial use for hospitals and teams caring for COVID-19 patients. Additionally, the awareness of the potential for overuse of emerging COVID-19 therapies, often based on limited data, drove institutional guidelines to recommend use of investigational COVID-19 therapies only in the context of a clinical trial.

This study has several limitations. Early on, reduced COVID-19 test availability and concerns over reduced sensitivity of RT-PCR,^39^ may have caused COVID-19 patient misclassification and biased findings toward the null hypothesis. Comorbid conditions were extracted from ICD-10 coding and were not verified though manual chart review, which may have impacted analyses of association with antimicrobial receipt. The relatively small COVID-19 patient population may have reduced power to detect significant associations between antimicrobial use and patient characteristics. Larger studies of antimicrobial use in COVID-19 patients are needed. Antimicrobial use was evaluated only in hospitalized patients cared for by internal medicine and MICU teams to reduce confounding from unmeasured differences in team structures and specialty-specific clinical approaches. Antimicrobial use changes for COVID-19 patients on other teams were not evaluated; however, those teams received COVID-19 huddle guidance, and findings would likely be similar. Antimicrobial use changes for COVID-19 outpatients were not evaluated; further study is needed. Because of the short COVID-19 pre-intervention period, it is not clear to what extent, if any, antimicrobial use may have decreased over time with increasing comfort level of providers caring for COVID-19 patients as well as increased use of VUMC COVID-19 guidelines. However, the rate of change would likely be slower. Antimicrobial use evaluation was limited to 14 days after hospitalization or COVID-19 positive test results, whichever came later, to restrict analysis to the period most likely to be associated with initial COVID-19 presentation. Inclusion of all antibiotics over a hospitalization may have biased findings to report an association with specific patient characteristics. Patients with prolonged stays might be more likely to have specific comorbidities and be more likely to receive antibiotics for healthcare-associated infections unrelated to COVID-19. As a result, our findings may underreport total antimicrobial use and potential associations for patients. We were unable to evaluate potential harms associated with antimicrobial use reduction, which will be an important balancing measure in evaluation of future antimicrobial stewardship interventions. A daily, multispecialty COVID-19 huddle is an intensive intervention, which may limit generalizability. However, the principle of routine antimicrobial use review and feedback for providers of COVID-19 patients who, evidence suggests, have low bacterial coinfection, can be adapted to many healthcare settings.

The interconnected antimicrobial resistance and COVID-19 pandemics represent threats to individual and public health with potential for mutual exacerbation. Beyond the danger of the COVID-19 pandemic promoting antimicrobial resistance, there are broader concerns for downstream collateral damage to previous gains made through antimicrobial development policies and stewardship.^14,33^ This study is the first to describe significant reductions in team-based antimicrobial use after COVID-19 in the context of an institutional systems-based approach, utilizing infectious diseases and stewardship guidance for COVID-19 providers. This may be a strategy to mitigate unnecessary antimicrobial use and optimize COVID-19 patient care moving forward.

The COVID-19 treatment and diagnostic landscape is evolving. Clinicians need antimicrobial use descriptive data to inform antimicrobial stewardship interventions to reduce inappropriate antibiotic prescribing for COVID-19 patients. Including antimicrobial stewardship expertise in a multispecialty COVID-19 management plan may reduce team-based antimicrobial use. Further study is needed to explore COVID-19 patient factors associated with antibiotic receipts, to assess secondary bacterial infection risk, and to characterize the effects of the COVID-19 pandemic on antimicrobial use across the healthcare spectrum.

## Supplementary material

For supplementary material accompanying this paper visit https://doi.org/10.1017/ice.2020.1291. click here to view supplementary material
