# Supplementary figures and images for: Changes in antimicrobial utilization during the coronavirus disease 2019 (COVID-19) pandemic after implementation of a multispecialty clinical guidance team
Source: Infect Control Hosp Epidemiol. 2020 Oct 26:1–7. doi: 10.1017/ice.2020.1291 (PMC7683821; doi:10.1017/ice.2020.1291)

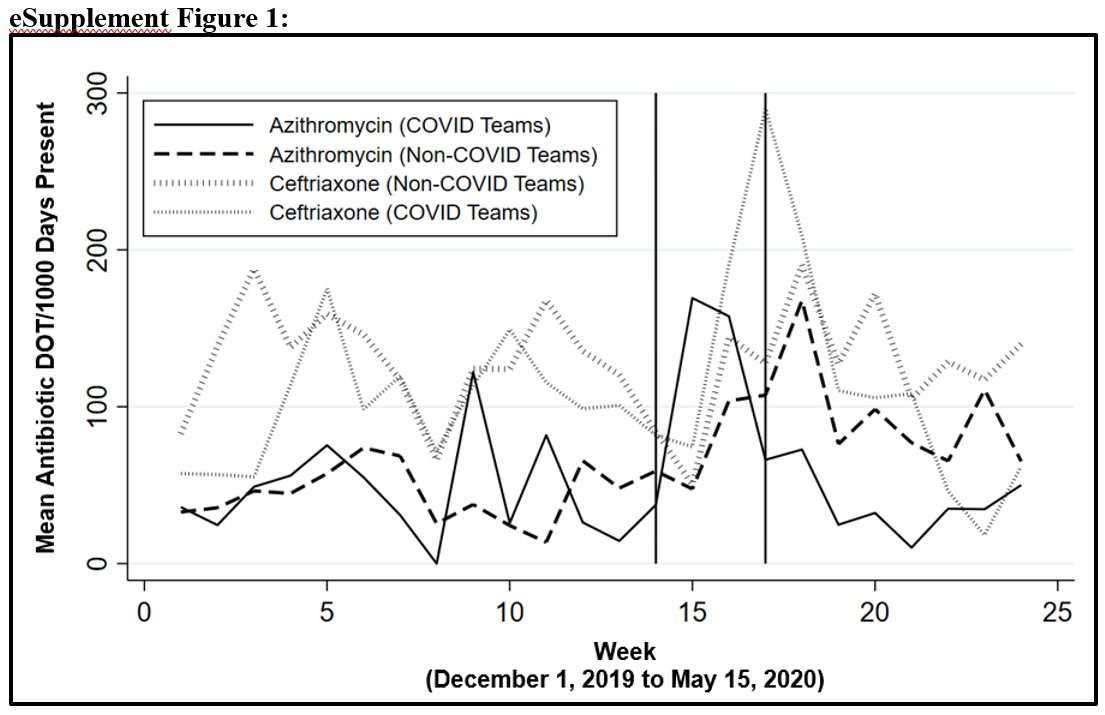

Supplement: Supplementary file 1 [file S0899823X2001291Xsup.zip › S0899823X2001291Xsup001.tif]

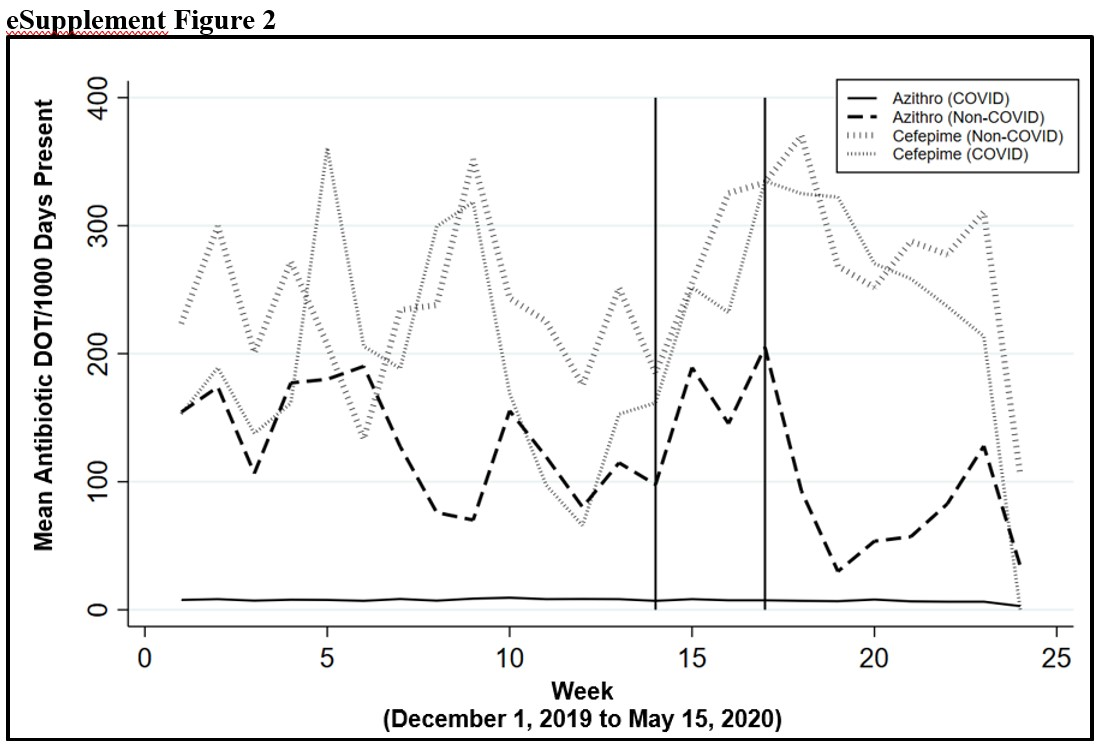

Supplement: Supplementary file 1 [file S0899823X2001291Xsup.zip › S0899823X2001291Xsup002.tif]
